# Quality of Life and Related Factors among HIV-Positive Spouses from Serodiscordant Couples under Antiretroviral Therapy in Henan Province, China

**DOI:** 10.1371/journal.pone.0021839

**Published:** 2011-06-30

**Authors:** Duo Shan, Zeng Ge, Shuai Ming, Lan Wang, Michael Sante, Wensheng He, Jianping Zhou, Shanglong Liu, Lu Wang

**Affiliations:** 1 National Center for AIDS/STD Control & Prevention, Chinese Center for Disease Control & Prevention, Beijing, China; 2 Fuwai Hospital and Cardiovascular Institute, Chinese Academy of Medical Sciences and Peking Union Medical College, Beijing, China; 3 Zhumadian Center for Disease Control and Prevention, Zhumadian City, Henan Province, China; 4 Tongji Medical College, Huazhong University of Science and Technology, Wuhan, China; University of Cape Town, South Africa

## Abstract

**Objective:**

To describe the quality of life and related factors in HIV-positive spouses undergoing ART from discordant couples.

**Methods:**

A cross-sectional study was conducted among 1,009 HIV-positive spouses from serodiscordant couples in Zhumadian, Henan Province, between October 1, 2008 and March 31, 2009. HIV-positive spouses were interviewed by local health professionals. Quality of life was evaluated by WHOQOL (Chinese Version). A multiple linear regression model was used to analyze the related factors.

**Results:**

The majority of subjects were female (56.39%), had received a high school education (44%), were of Han ethnicity (98.41%), and were farmers (90.09%); the median time period of receiving ART was 3.92 years. The physical, psychological, social, and environmental QOL scores of the subjects were 12.91±1.95, 12.35±1.80, 13.96±2.43, and 12.45±1.91 respectively. The multiple linear regression model identified the physical domain related factors to be CD4 count, educational level, and occupation; psychological domain related factors include age, educational level, and reported STD symptom; social domain related factors included education level; and environmental domain related factors included education level, reported STD symptoms, and occupation.

**Conclusion:**

Being younger, a farmer, having a lower level of education, a reported STD symptom, or lower CD4 count, could decrease one's quality of life, suggesting that the use of blanket ART programs alone may not necessarily improve quality of life. Subjects received lower scores in the psychological domain, suggesting that psychological intervention may also need to be strengthened.

## Introduction

Since the discovery of the first AIDS case in the United States in 1981, HIV/AIDS has become a major worldwide health problem [1], [2], [3]. There were an estimated 740,000 people living with HIV/AIDS in China by the end of 2009 [4]. Infection with HIV causes progressive immunodeficiency resulting in a variety of opportunistic infections, which could cause physical and psychological damage and decreased quality of life [5], [6]. Serodiscordant couples, defined as couples in which one partner is HIV-positive and the other is HIV-negative, have the risk of HIV transmission by heterosexual intercourse, which could have a negative impact on their physical and psychological health and thus decrease their quality of life (QOL)[7], [8]. HIV-infected spouses from serodiscordant couples face multiple challenges, including the stress of sexual transmission [9], financial pressures, and coping with HIV-related stigma, all of which may have negative influence on their quality of life [10]. According to census statistics, people living with HIV in Zhumadian City account for almost 1/3 of all HIV cases in Henan province. Most patients in this area became infected through the sale of plasma between 1992 and 1995 [11], [12]. In 2003, Antiretroviral Therapy (ART) was introduced for HIV-infected individuals and by 2005 most of the HIV-infected spouses were regularly treated in Henan, China. The effectiveness of ART could lead to more HIV serodiscordant couples, as ART prolongs the lives of AIDS patients. ART for AIDS patients has also shifted the perception of HIV/AIDS from a fatal infection to a chronic yet manageable infection, which makes the impact of ART on the overall quality of life among AIDS patients a major issue with subsequent implications for the long-term care of HIV-positive individuals, their social adjustment to the illness, and their interaction with health care providers. Meanwhile, patients undergoing ART with a history of 7 years might experience side effects of the ART regimen and drug resistance; this, along with having to take medicine daily, could also bring trouble to patients [13], [14], [15]. The effectiveness of ART has often been measured by reduction in mortality, opportunistic infection rates, or side effects; however, evaluation on quality of life for HIV-positive spouses under ART from serodiscordant couples has not been well documented in China, and doing so would have significant importance [13]. Identifying factors that diminish QOL in HIV-positive spouses is an important step towards improving QOL in this population. This cross-sectional study will describe quality of life and related factors among HIV-positive spouses from serodiscordant couples under ART in Zhumadian City, Henan province, in order to assess the impact of socio-demographic and AIDS-related variables on quality of life and to facilitate ART and social care programs.

## Methods

### Ethics Statement

The study received approval from the institutional review board of the China CDC's National Center for AIDS/STD Control & Prevention. All participants were provided with free counseling and evaluation for treatment. We obtained written informed consent from all participants involved in our study who signed their own names on the informed consent form included in our questionnaires. We protected the privacy of individuals in processing personal data and maintained confidentiality of individual records and accounts. The ethics committees approved this consent procedure.

### Study Setting

This cross-sectional study was conducted among HIV-positive spouses in HIV serodiscordant couples between October 2008 and May 2009 in Zhumadian City, Henan Province, China. HIV discordant couples were identified from the HIV-positive database administered by the Zhumadian City Center for Disease Control and Prevention. Local staff enrolled study participants from county hospitals, community health centers, or from their own residence. The inclusion criteria were as follows: 1) HIV-positive people living with an HIV-negative partner; 2) Be in a stable marriage (having not separated nor divorced from their spouse); 3) Be an AIDS patient undergoing ART; 4) Having participated voluntarily and been provided informed consent.

A standardized questionnaire was administered by trained local healthcare workers among HIV-positive spouses between October 2008 and May 2009. The patients were required to rate their situation as of two weeks prior to the interview. Survey questions included information on demographic characteristics, sexual activity, condom use, quality of life, STI (sexually transmitted infections) history, as well as medical history. The last recorded CD4+ cell count of HIV-positive spouses was obtained from the China CDC national case reporting database.

### Qol Assessment Instrument

QOL was assessed using the WHOQOL-BREF (Chinese version) [15], [16] which is an easy to use instrument developed by WHO for the purpose of evaluating health-related QOL and making cross-cultural comparisons [17]. WHOQOL-BREF was used for this study based on the following reasons: (1) Studies showed that a variety of instruments have been utilized to evaluate QOL of people living with HIV/AIDS, including the Medical Outcome Study HIV Health Survey (MOS-HIV), the Quality of Well-Being, the HIV-QL31, the SF-36, the Euro-QOL, and WHOQOL-HIV Survey [18], [19], [20], [21], [22], [23]. Most of the instruments were developed in the context of Western culture and may not be applicable to patients from Asian countries that have different cultural backgrounds. The WHOQOL-BREF quality of life assessment was developed by the WHOQOL Group with fifteen international field centers, including China, so that it could be generally applicable across cultures; (2) The internal reliability and validity of WHOQOL-BREF has been tested rigorously using Rasch and Item Response Theory Models and by the WHOQOL Group. Analyses of internal consistency, item-total correlations, discriminant validity, and construct validity through confirmatory factor analysis indicate that the WHOQOL-BREF has good to excellent psychometric properties of reliability and performs well in preliminary validity tests. (3) It has been used in a number of studies and different populations, such as early adolescence, the general population, and patients with HIV infection [24], [25], [26], [27], [28]. (4) It is quicker (only takes 8-10 minutes) and convenient to use in larger research; (5) the WHOQOL (Chinese version) is readily available and has been successfully used in the evaluation of QOL of HIV/AIDS individuals in Chinese population and has proved to be a reliable and useful tool [29].

The WHOQOL-BREF consists of 26 items, including 24 items for four domains (physical, psychological, social, and environmental), one item for general quality of life, and one item for health-related quality of life [14]. The physical domain includes three facets: pain and discomfort; energy and fatigue; and sleep and rest. The psychological domain includes five facets: positive feelings; negative feelings; learning and concentration; bodily image; and self-esteem. The social domain includes three facets: personal relationships; practical social support; and sexual activity. The environmental domain includes five facets: financial resources; healthcare availability; opportunities for acquiring new information and skills; opportunities for leisure; and transport. Each facet includes between two and eight items. Each item uses a Likert-type five-point scale, with a higher score indicating a better QOL. The equation suggested by WHO (World Health Organization, WHOQOL User Manua1, WHO, Geneva. 1998) was applied to the overall estimation of each domain. Because the numbers of items were different for each domain, the domain scores were calculated by multiplying the average of the scores of all items in the domain by 4. Thus, the domain scores would have the same range from 4 to 20, making it convenient to compare the scores in different domains, populations, studies, or regions.

### Statistical Analysis

Statistical analysis was performed using SPSS software package version 15.0 (SPSS Inc., Illinois, USA). The internal consistency reliability was assessed using Cronbach's coefficient α; a Cronbach's coefficient α of 0.7 or above was considered acceptable. Pearson's coefficient was performed to examine the correlation between four domains with total scores. Percents and frequencies were used for categorical variables, and means, standard deviations, and range (minimum and maximum) were calculated for continuous variables. As used in many other studies [28], [29], [30], [31], Student's t-test and ANOVA were performed for comparing and testing domain scores between categories or among categories, respectively. Univariate linear regression models were run to assess the unadjusted relationship between the scores of four domains and specified covariates of interest for all subjects. A multivariate regression model was used in which dependent variables were the scores of the four domains and independent variables included social demographic, migration worker status after becoming HIV-positive, sexual intercourse status in the past three months, and medical history, in order to assess the adjusted relationship between quality of life and these independent variables. Covariates with a p-value <0.10 from the univariate analysis were entered into a full multivariate regression model and stepwise selection was used to include significant covariates. All hypothesis testing was based on 2-sided tests with an alpha level of 0.05.

## Results

### Social-demographic Characteristics

Between October 1, 2008 and March 31, 2009, 1,009 HIV-positive spouses from serodiscordant couples completed the questionnaire. Of these, 440 (43.61%) were men and 569 (56.39%) were women. The average age of those who participated in the study was 43.55±6.86 years (a range of 29–60 years old). 993 (98.41%) were of Han ethnicity and 16 (0.4%) were of Hui or Chaoxian ethnicity. 909 (90.09%) were farmers and 100 (9.91%) had other occupations. 113 were illiterate, 434 received education for less than or equal to 6 years, 444 received education for 7–9 years, and 18 received education for more than 10 years. 551 (54.61%) had an experience as a migrant worker after having been confirmed as HIV-positive, 458 (45.39%) had no such experience. 839 (83.15%) had sexual intercourse in the past three months and 170 (16.85%) had no sexual intercourse in the past three months (See Table 1).

**Table 1:** Demographic Information, mean scores and standard deviations of four domains of Chinese WHOQOL-BREF (n = 1009).

Variables	N (%)	Physical ( 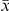 ±*s*)	Psychological ( 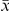 ±*s*)	Social ( 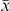 ±*s*)	Environmental ( 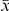 ±*s*)
Total scores of QOL		12.91±1.95	12.35±1.80	13.96±2.43	12.45±1.91
Sex					
Male	440 (43.61)	12.80±1.96	12.23±1.80	13.80±2.41	12.36±1.80
Female	569 (56.39)	13.00±1.93	12.44±1.80	14.07±2.45	12.53±1.99
Age					
<40	351 (34.79)	12.85±1.95	12.24±1.83	13.84±2.50	12.34±1.88
40–49	444 (44.00)	12.90±1.91	12.33±1.75	13.93±2.36	12.46±1.85
50-	214 (21.21)	13.03±2.01	12.57±1.85	14.22±2.46	12.63±2.07
Ethnicity					
Han	993 (98.41)	12.91±1.95	12.36±1.80^a^	13.97±2.44	12.46±1.91
Others	16 (1.59)	12.50±2.01	11.46±1.33	13.58±2.02	12.03±1.56
Occupation					
Farmer	909 (90.09)	12.83±1.97^a^	12.32±1.84	13.89±2.47^a^	12.39±1.95^a^
Other	100 (9.81)	13.60±1.61	12.57±1.39	14.59±2.02	13.01±1.29
Educational level					
0 years	113 (11.20)	13.10±2.15	12.34±1.75^b^	14.34±2.35^b^	12.50±2.09
≤6 years	434 (43.01)	12.83±1.89	12.45±1.75	14.06±2.39	12.44±1.93
7–9 years	444 (44.00)	12.06±3.40	10.89±3.35	12.85±2.42	11.64±2.20
≥10 years	18 (1.78)	12.91±1.95	12.35±1.80	13.96±2.43	12.45±1.91
Migrate worker after HIV-positive					
Yes	551 (54.61)	12.94±1.84	12.48±1.75^a^	13.99±2.39	12.56±1.90^a^
No	458 (45.39)	12.88±2.07	12.19±1.85	13.92±2.49	12.32±1.90
Sexual Intercourse in past 3 months					
Yes	839 (83.15)	12.94±1.89	12.37±1.72	13.94±2.38	12.45±1.86
No	170 (16.85)	12.76±2.18	12.24±2.17	14.08±2.69	12.48±2.12
Reported STD symptom					
Yes	25 (2.48)	12.66±2.49	12.23±1.63^a^	13.44±1.92	11.54±1.85^a^
No	984 (97.52)	12.92±1.93	12.38±1.80	13.97±2.44	12.48±1.90
Switched ART regimen^c^					
Yes	211 (20.91)	12.75±2.19	12.26±2.10	13.94±2.58	12.47±1.99
No	798 (79.09)	12.95±1.88	12.37±1.72	13.97±2.39	12.45±1.89
The length of ART( Median (years):3.92)					
<1 year	110 (10.90)	12.87±1.91	12.26±1.81	13.63±2.41	12.56±1.92
1–2 years	120 (11.89)	12.98±2.17	12.32±2.20	14.26±2.91	12.56±2.36
2–3 years	184 (18.24)	12.74±2.07	12.15±1.80	14.06±2.59	12.44±1.98
3–4 years	300 (29.73)	12.94±1.78	12.32±1.60	14.13±2.22	12.41±1.67
4–5 years	220 (21.80)	13.04±2.11	12.50±1.99	13.60±2.41	12.34±2.00
≥5 years	75 (7.43)	12.76±1.33	12.64±1.1.5	14.08±1.96	12.68±1.52
Most recent CD4 counts					
≤200	289 (28.64)	12.75±2.00	12.25±1.84	13.96±2.51	12.43±1.94
201–350	278 (27.55)	12.86±1.91	12.32±1.79	13.88±2.47	12.37±1.91
>350	442 (43.81)	13.05±1.93	12.43±1.79	14.02±2.36	12.52±1.88

a t-test, *p*<0.05; ^b^ one-way ANOVA p<0.05; ^c^First-line scheme: AZT/D4T+3TC + EFV/NVP, alternative scheme: AZT/D4T+3TC + IDV, DDI+D4T + EFV/NVP or AZT + DDI + EFV/NVP. As of the date of survey, patients undergoing ART who has ever changed from the above first-line scheme to alternative scheme were classified as ART regimen switched.

### Medical History

Table 1 shows that 97.52% of respondents had no reported STD symptom in the past, while 2.48% had reported STD symptoms in the past. The median duration of HIV/AIDS patients who had received ART was 3.92 years, 10.90% for less than 1 year, 11.89% for more than 1 year but less than 2 years, 18.24% for more than 2 years but less than 3 years, 29.73% for more than 3 years but less than 4 years, 21.80% for more than 4 years but less than 5 years, and 7.43% for more than 5 years. 20.91% of the respondents had switched ART regimens and 79.09% had never switched. Table 1 shows the differences of the mean quality of life scores in the physical, psychological, social, and environmental domains among HIV-positive spouses from serodiscordant couples during ART treatment. In general, scores of quality of life in the social domain were the highest and scores in the psychological domain were the lowest among the four domains. Scores of quality of life in the four domains among those who underwent ART for 1–2 years were relatively higher than other lengths of ART, while scores in the social and environmental domains among those who underwent ART for 4–5 years were the lowest. The median CD4 count of those who underwent ART was 313. 28.64% of the respondents had a CD4 count of less than 200, 27.55% had a CD4 count of more than 201 but less than 350, and 43.81% had a CD4 count of more than 351.

### Quality of Life

HIV-positive spouses had a mean score of 12.91±1.95 in the physical domain, 12.35±1.80 in the psychological domain, 13.96±2.43 in the social domain, and 12.45±1.91 in the environmental domain. There was no statistical difference in the quality of life scores between men and women. Although the quality of life score increased with age, there was no statistical difference among age groups. Ethnic difference had a statistical significance in the psychological domain, with Han receiving higher scores than others. There was no statistical difference in the quality of life scores of the other domains. The physical, social, and environmental domains had statistical differences between farmers and other occupations, with farmers receiving lower scores than other groups. The psychological and social domains contained statistical differences among age groups, and the group with an educational level between 7–9 years received the lowest scores. The psychological and environmental domains contained statistical differences between groups of migrant workers who had been confirmed as HIV-positive. Those with previous experience as a migrant worker received higher scores than those with no experience. The psychological and environmental domains were statistically different between the group that reported STD symptoms and the group that reported no symptoms; the group that reported no STD symptoms received higher scores than those who reported STD symptoms. There was no statistical difference in the quality of life scores of those who had switched ART regimens and of those who had not switched ART regimens. The median length of ART is 3.94 years. There was no statistical difference in the quality of life scores among different CD4 count groups, but the scores in the physical and psychological domains did increase with increased CD4 counts.

### Results of Multiple Stepwise Regression Analysis

Table 2 shows four distinct models for quality of life. For quality of life in the physical domain, the last recorded CD4 count and educational level are associated factors. In the psychological domain, age, educational level, reported STD symptoms, and migrant worker experience are associated factors. In the social domain, education level and occupation are associated factors. In the environmental domain, education level, reported STD symptoms, migrant worker experience, and occupation are associated factors.

**Table 2:** Results of multiple stepwise regression analysis for QOL.

Dependent variable	Independent variable	Coefficients	Std. Error	t	*p* value
QOL-physical domain	CD4 counts				
	201–350	0.113	0.163	0.69	0.49
	>350	0.262	0.122	2.14	0.03^a^
	Educational level				
	≤6 years	−0.168	0.124	−1.35	0.18
	7–9 years	−0.121	0.226	−0.54	0.59
	≥10 years	−1.026	0.460	−2.23	0.03^ b^
	Occupation	0.814	0.204	4.00	<0.01^c^
QOL-psychological domain	Age				
	40–49	0.014	0.129	0.11	0.92
	≥50	0.285	0.137	2.08	0.04^d^
	Educational level				
	≤6 years	0.195	0.199	0.98	0.33
	7–9 years	0.097	0.208	0.47	0.64
	≥10 years	−1.458	0.423	−3.45	<0.01^b^
	Reported STD symptom	−1.054	0.361	−2.92	<0.01^e^
	Migrate Worker	0.252	0.113	2.23	0.03^f^
QOL-social domain	Educational level				
	≤6 years	−0.181	0.269	−0.68	0.50
	7–9 years	−0.225	0.159	−1.41	0.16
	≥10 years	−2.213	0.575	−3.85	<0.01^b^
	Occupation	0.773	0.255	3.04	<0.01
QOL-enviromental domain	Educational level				
	≤6 years	0.025	0.122	0.20	0.84
	7–9 years	0.028	0.222	0.13	0.90
	≥10 years	−0.920	0.451	−2.04	0.04^b^
	Reported STD symptom	−0.838	0.384	−2.18	0.03^e^
	Migrate Worker	0.278	0.121	2.30	0.02^f^
	Occupation	0.709	0.202	3.52	<0.01^c^

Reference group: ^a^Compared with ≤200 *p*<0.05; ^b^Compared with 0 year *p*<0.05;

c Compared with farmer *p*<0.05; ^d^Compared with <40 *p*<0.05; ^e^Compared with no reported STD symptom *p*<0.05; ^f^Compared with no migrate worker *p*<0.05.

## Discussion

Our study of 1,009 HIV-positive spouses from HIV discordant couples undergoing ART in Zhumadian City, Henan Province, using the Chinese WHOQOL-BREF system to assess their quality of life, was the first and largest of its kind in China. If one of the main purposes of health care is to improve QOL, then it is important to understand not only QOL status, but also its influential factors of HIV-infected persons. In fact, a major finding of our study was a mean physical domain score of 12.91±1.95, psychological score of 12.35±1.80, social score of 13.96±2.43, and environmental score of 12.45±1.91. Our subjects got a higher score in the social domain than the average level of the population in general, which might be associated with the larger number of HIV-positive individuals in Zhumadian and the health education to decrease stigma and discrimination in this population [32]. The lowest score of the subjects was in the psychological domain, which is lower than the average level of the population in general (See Table 3) [15].

**Table 3:** The comparison of mean and SD among different populations.

Subdomains	Results in our study( 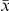 ±s)	Domain general population( 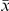 ±s) a,b,c	t	*p* value
Physical	12.91±1.95	15.8±2.9	−47.16	<0.001
Psychological	12.35±1.80	14.3±2.5	−34.41	<0.001
Social	13.96±2.43	13.7±3.0	3.40	= 0.001
Environmental	12.45±1.91	13.2±2.4	−12.42	<0.001

a S.M.Skevington, M.Lotfy,K.A.O'Connell.(2004)The World Health Organization's WHOQOL-BREF quality of life assessment: sychometric properties and results of the international field trial A Report from the WHOQOL Group.Quality of life research,13:299–310.

b The WHOQOL Group. The world health organisation quality of life assessment (WHOQOL): position paper from the World Health Organisation. Social Science and Medicine, 1995, 41, 1403–1409.

c The WHOQOL Group. The world health organisation quality of life assessment (WHOQOL): development and general psychometric properties. Soc Sci Med, 1998, 46: 1569–1585.

Among socio-demographic variables, age, ethnicity, occupation, and education level were associated with quality of life. Although findings from other studies demonstrated a relationship between gender and QOL [30], [33], [34], our study found no statistical difference between male and female groups in any domain. The reasons might be associated with the subjects and the tools used for measurement. An older age was associated with a high score in the psychological domain, which was consistent with the study conducted in Tehran, Iran [30]. Farmers had lower scores in all four domains possibly because they generally had lower living standards in the rural areas and many of them had fewer opportunities for social contact. The reason for patients with an education of 7–9 years receiving the lowest scores for quality of life is still not well understood. More detailed study is needed.

Among medical history variables, HIV-infected persons with STD symptoms had worse health conditions, which might explain why their QOL was worse. As in other studies, we found that a lower CD4 cell count was associated with a lower quality of life, which was consistent with other studies [17], [30], [35]. That patients with a higher CD4 cell level had a higher quality of life suggests that ART is good for improving CD4 cell levels, physical symptom relief, and recovered immunity [36], [37]. Although viral loads have been more directly correlated with quality of life, we were unable to obtain viral load data from these rural areas because of problems with infrastructure and human resource capacity. There is a slow decrease in the first 3 years of receiving ART and then a slow increase as ART continues; however, we do not observe any difference in the four domains of QOL among HIV-infected persons who have received ART for different lengths of time.

There may have been a social desirability bias because many of the respondents might have underreported their STD symptoms. The adherence data for those under ART was not attained because reliable adherence data was not tracked by local health centers where nearly all of the patients received treatment. Future studies on this topic may incorporate more detailed questions on drug adherence and quantify drug resistance. Our findings suggested that ART may have an impact on quality of life in varying degrees among HIV-positive spouses in China, and that ART is not sufficient to improve the quality of life among this population. To be most effective, ART programs in these areas should be combined with HIV prevention intervention that emphasizes psychological intervention [38], active treatment of STDs, and care and support services.
